# Drivers of Household Recycling Behavior in the City of Johannesburg, South Africa

**DOI:** 10.3390/ijerph19106229

**Published:** 2022-05-20

**Authors:** Dorothea Christina Schoeman, Isaac Tebogo Rampedi

**Affiliations:** Department of Geography, Environmental Management & Energy Studies, University of Johannesburg, P.O. Box 524, Auckland Park, Johannesburg 2006, South Africa; theas@uj.ac.za

**Keywords:** household recycling behavior, socio-demographical variables, recycling drivers, factor analysis, recycling benefits, perceived control, situational factors

## Abstract

This paper has assessed the relationship between recycling behavior and socio-demographic variables for households in Johannesburg, South Africa. The research also identified the underlying driving factors that motivate recyclers to separate their household waste for recycling. These objectives were addressed by means of a quantitative survey research design as well as descriptive and inferential statistical methods. Based on the results, the statements that represented attitudes, subjective norms, perceived control, moral norms, situational factors, outcomes, and consequences of recycling were highly agreed to by respondents. Three factors influencing household recycling behavior were identified, namely, recycling benefits, perceived control, and situational variables. The recycling benefits construct explained 45.6% of the variance, followed by the perceived control construct (12%) and the situational variables construct explained 11% of the variance. However, the results of the EFA and multiple regression identified the situational variable as the greatest single driver of household participation in recycling. It is therefore important to overcome situational barriers that the residents of Johannesburg are currently facing if the current household waste separating program is to become successful. This would require simplifying the process of household recycling by providing appropriate knowledge, infrastructure, and the space for waste sorting and its collection.

## 1. Introduction

Based on the 2018 State of Waste Report, during the year 2017, about 55 million tons of waste was generated in South Africa and only 11% was diverted away from landfilling [1]. This is an unsustainable trend especially as waste generation rates are bound to increase further in South Africa given the rapidly increasing population and economic growth rates as well as urbanization rates. There is, therefore, an urgent need to develop and implement efficient strategies to divert wastes away from landfill sites so that their assimilative capacity and airspaces are not exceeded [1]. Such strategies would require far-reaching measures to encourage waste minimization behavior in society by means of resource recovery, reuse, and recycling. As a result, South Africa has witnessed increased promulgation of environmental legislation, regulation, and various declarations to help reduce environmental degradation, high waste generation rates, and associated landfilling. For instance, two of the key principles underpinning the 2020 National Waste Management Strategy (NWMS) are waste minimization and using waste as a resource through re-use, recycling, treatment, and recovery. Where waste cannot be prevented, an outcome of the NWMS is to divert 40% of such wastes from landfills within five years, 55% within 10 years, and at least 70% waste diversion within 15 years [1].

Like many other previous declarations, the achievement of such waste prevention or increased waste reduction goals remains unrealistic as long as there is a disconnection in the understanding of waste recycling behavior and its determinants amongst different stakeholders in the South African society. For instance, in the 2014 General Household Survey involving 25,363 respondents in South Africa, it was found that nearly 70% of respondents in urban areas were not participating in any household recycling practices mainly because there were no appropriate recycling bins [2]. Moreover, only 3% of urban residents recycled their waste on a regular basis in 2010 because of deficiencies in raising public awareness to change mindsets [3]. Similarly, a study conducted by Strydom [4] indicated that non-recycling households (74% out of 2004 respondents) were constrained by a lack of time and relevant knowledge to segregate their recyclable waste items. Furthermore, stakeholders such as businesses in South Africa have raised concerns that some of the legislation promulgated has actually created major barriers to the growth of the national recycling economy [5,6]. 

Recycling features in the waste minimization side of the solid waste management hierarchy, and therefore, it can contribute to reduced natural resource consumption and increased re-utilization of salvageable wastes to satisfy human needs. Recycling is also regarded as one form of pro-environmental behavior that can limit the negative impacts of human activities on the natural environment and human health [7,8,9]. However, engaging and involving various stakeholders to sort or segregate waste items at their source of generation is not an easy feat and require numerous interventions such as raising awareness levels, providing the necessary infrastructure, and understanding the critical factors to encourage recycling behavior [10].

In the pursuit of understanding recycling behavior and its determinants, various theories and models have been developed in the literature, of which only two will be briefly explained to help contextualize the present research. According to the Schwartz altruism model, personal and social concerns combine to influence the altruistic behavior of individuals [11]. Schwartz [12] defined altruistic motivation as “intentions or purposes to benefit another as an expression of internal values, without regard for the network of social and material reinforcements”. For instance, if a recycling program is introduced, a person may first use social norms to decide whether or not to participate. Therefore, willingness to engage in environmental conservation may be explained in terms of prevailing social norms [13,14]. Such norms may stand alone or be combined with internalized personal norms from related activities to determine what would constitute proper behavior. If a person decides to recycle, this norm may be internalized, thus becoming a personal norm. However, the next important step in this model is the linkage between personal norms and behavior. A person may internalize the norms, but then fail to act based on these norms [15]. It is only when a person defines these norms as relevant and applicable to a situation, will norms begin to play a role in behavior [11]. Furthermore, other moderating variables in this model are awareness of consequences and the ascription of responsibility of consequences. It is therefore important to understand the process a person undergoes from altruistic social norms and then translate them into individual behavior. Although mixed results have been generated by the application of this model to predict behavior, a study conducted by Khan et al. [16] has indicated how various consumers’ dimensions and their attitudes led to different types of recycling behavior when it comes to waste disposal management in Karachi (Pakistan). This conclusion is in line with a case study conducted by Miliute-Plepiene et al. [10], which revealed that the activation of moral norms was one of the most important factors for household recycling outcomes in Lithuania and Sweden.

By contrast, to predict behavior on the basis of the theory of planned behavior (TPB) requires different conditions to be satisfied in terms of intentions, subjective norms, attitudes, and perceived behavioral controls. For example, the measures of intention and the perceived behavioral control must be compatible with the behavior that the theory is predicting [17]. In a recycling context, if the TPB is to predict participation in recycling, it must assess ‘to participate in recycling’ and not a general intention such as ‘to engage in pro-environmental behaviour’. According to Azjen [17], ‘an essential prerequisite for assessing a person′s actual control is a good understanding of the various internal factors (skills, knowledge, physical stamina, and intelligence) and external factors (legal barriers, money, equipment, and cooperation by others) that are needed to perform the behavior or that can interfere with its performance’ [17]. Lastly, normative beliefs contribute to the general perceived social pressure (i.e., subjective norms) to engage or participate in the behavior. Nonetheless, several criticisms have been leveled against the TPB, of which one of them is that the theory assumes contiguity between intention and behavior just as its predecessor, namely, the theory of reasoned action [11]. Another problem identified in the literature is that the TPB only introduces one additional variable, while there is evidence that other factors add predictive power to recycling behavior. In reply, both Azjen [17] as well as Azjen and Fishbein [18] clarified that the TPB does not specify what particular perceived behavioral controls are to be associated with a specific behavior and that it is open to the inclusion of additional variables. It is therefore not surprising that numerous studies have explored recycling behavior by considering a wide variety of variables that influence recycling behavior [17]. To name a few: Oztekin et al. [19] investigated gender perspectives in predicting recycling behavior; Jekria and Daud [20] connected environmental concern with recycling behavior; and Amini et al. [21] investigated the influence of reward and penalty on household’s recycling intention. Even so, the research carried out by Latif et al. [22] and Ma et al. [23] showed that situational factors are also a significant predictor of recycling behavior and thus are in agreement with the results of Schultz et al. [24] derived from a review of personal and situational variables in recycling. In this way, the TPB has become one of the most applied theories in modeling recycling behavior while other studies are building on it, thus introducing other moderating variables. 

This paper has assessed the relationship between recycling behavior and the socio-demographic variables of gender, age, education, and income amongst households in the City of Johannesburg (CoJ), South Africa. The research also identified the underlying driving factors that motivate recyclers to separate their household waste for recycling. This research problem is worthy of an investigation for various reasons. Firstly, available evidence show that the CoJ has only about 5 years of remaining landfill space [25]. Such a shrinking landfill space will not be able to accommodate more incoming wastes, especially as the amounts of wastes generated are expected to increase by 13% annually [25], with only 7% of them being diverted away from landfilling through recycling and composting [26]. Therefore, to increase recycling rates in the CoJ and help to divert wastes away from landfills, the Separation at Source (S@S) program was implemented in 2009 at one depot and later to nine other depots, the goal being to encourage communities to participate in recycling [27]. Although there was a gradual increase in the recycling participation rates, recent rates have declined to as low as 19.9% [27]. Such a state of affairs is environmentally untenable and is not in line with the Sustainable Development Goal No. 11 of the United Nations which seeks to render cities and other human settlements more resilient and sustainable. More specifically, Target No. 11.6 of this goal aims to reduce the adverse per capita environmental impact of cities, including paying special attention to their air quality and municipal waste management issues [28]. Secondly, the identified research problem is justified in light of the shortcomings seen in the previous waste management literature in South Africa. For instance, other than the research conducted by Schoeman and Schmidt [29] which investigated recycling in only three suburbs of Johannesburg, no other study has investigated the drivers of recycling behavior in South Africa’s largest city—Johannesburg—although some studies [2,4,30] investigated it for the whole of South Africa. Furthermore, Du Toit et al. [31] investigated socio-spatial factors for household recycling for townhouses in Pretoria. Given this literature background, it is clear that the present research is the first study to contribute towards the identification of factors that drive participation in waste minimization in Johannesburg. It is also the first in-depth study of a metropolitan area in South Africa at a time when household waste separation participation is reported to be generally low among many jurisdictions [2,30,32]. Hence, the present study can be used as a comparative study for other metropolitan areas′ waste minimization efforts.

## 2. Study Area and Methods

### 2.1. Description of Study Area

**Figure 1:**
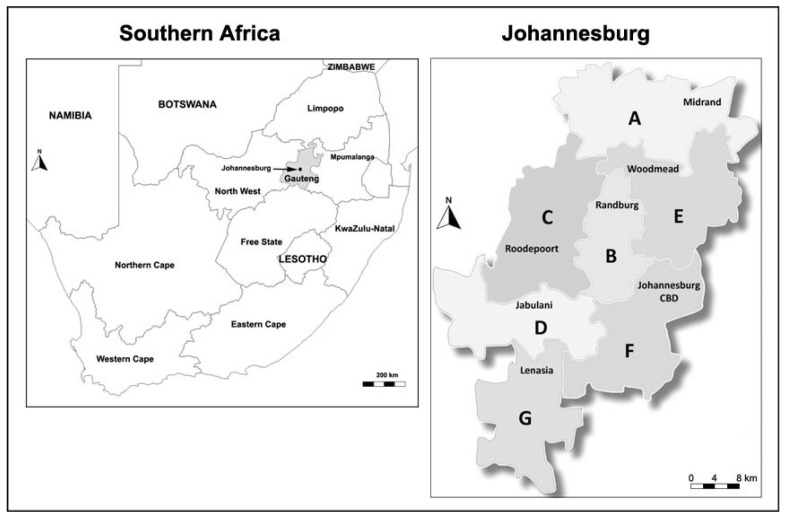
Geographical location of the study area in South Africa and the different regions of the CoJ.

The CoJ is the biggest metropolitan municipality in South Africa and is located in the Gauteng province of South Africa [29] (Figure 1). The population size is comprised of 5.5 million inhabitants and there are nearly 1.85 million households [33]. The land area is 1648 km^2^ and is divided into 7 different administrative regions with each one responsible for local community-based services.

The average income per capita is estimated to be R74 600 per year [34]. Johannesburg is classified as an upper-middle-income economy by the World Bank [35]. According to Madlalate [36], residential inequalities have survived the fall of apartheid and they are reinforced by economic inequalities. The poor residents in Johannesburg generally live in the low-income suburbs in the south and in the far northern parts of the city. 

Johannesburg is also considered to be the ‘economic hub’ of South Africa and people migrate to this city at a rate of approximately 3000 people per month [35]. During the 2008–2018 period, the CoJ’s population grew at an average percent of 2.91% per annum which exceeded the South African average of 1.56% per annum [33]. A unique geographical feature of Johannesburg is its relatively young population compared to the rest of South Africa. About 34.3% of the population is in the 14–35 years age group [35,37] and around 40% within the young working age of 24–44 years [33]. The reason for this ‘young’ population is that many young people migrate to Johannesburg in search of employment opportunities. However, there are high levels of youth unemployment with around 40% of the youth in the city not working in the formal or informal sector [35].

In 2018, the infrastructure backlog was in the order of R170 billion [35] and this left a tremendous strain on the CoJ’s operational infrastructure, including waste management services and facilities required for enhanced waste segregation at source. This constraint is compounded by the rapidly expanding population that puts additional pressure on service delivery in the city. It is for this reason that Muzenda et al. [30] as well as Karani and Jewasikiewitz [38] stated that local authorities in South Africa, and especially the CoJ, are struggling to implement an integrated solid waste management approach successfully. Waste management and related services fall under the Department of Environment and Infrastructure Services of the CoJ and are operated by the private contractor known as Pikitup.

According to the 2018–2019 Integrated Development Plan (IDP) of the City of Johannesburg, about 1.53 million (95%) households receive weekly refuse removal services while nearly 65,600 (4%) households are experiencing waste collection backlogs [39]. On the other hand, households receiving less than a weekly collection service are estimated to be 19,200 (1%). Their infrastructure consists of 12 depots across the seven regions of the CoJ, 6 sorting facilities and buy-back centers, 42 drop-off/garden sites, four operational landfill sites, and two closed landfill sites [33]. In addition, two privately owned landfill sites (Mooiplaats and Chloorkop) in the north of the CoJ are also used for waste disposal. Given the limited remaining capacity and airspace at the existing landfill sites, a waste separation at source (S@S) program was introduced in 2009, although participation rates by residents are very low (19%). Since households can make a significant contribution to divert wastes away from landfills, it is imperative to investigate recycling behavior, thus generating important insights that can contribute towards increased recycling participation while providing key knowledge to the local authority and Pikitup regarding the performance of the S@S program. 

### 2.2. Research Methods

To address the research aim, a quantitative survey design was adopted for the study, and a convenience sampling approach in line with the research conducted by Onwuegbuzie and Collins [40] as well as Collins [41] was used. According to them, convenience sampling entails selecting individuals that are conveniently available and willing to participate in the study. In determining the number (≈385) of study participants (or respondents), the confidence level was 95%, with a 0.5 standard deviation and a margin of error (confidence interval) of ±5%. 

The questionnaire was pretested with a smaller group of respondents and important lessons were generated. For example, the word ‘recycling’ was used in the questionnaire as the feedback from the pilot survey indicated that ‘recycling’ is a word that respondents are more familiar with than the correct phrase of ‘separating wastes’. Thus, the general public uses the word ‘recycling’ and they do not make the academic distinction between ‘recycling’ and ‘household waste separation’ for recycling purposes.

The final questionnaire was based on the recycling literature and studies that applied the theory of planned behavior and consisted of two sections. Section A was based on the demographic information and section B obtained information on recycling participation while others were tested using a five-point Likert scale ranging from ‘strongly disagree’ to ‘strongly agree’. Twenty-five (25) different statements regarding recycling were given to respondents to investigate their attitude, subjective norms, perceived control, moral norms, situational factors, outcomes, and consequences of recycling. The questionnaires were self-administered for self-reporting and participation in the study was voluntary. To this end, respondents were duly informed about the aim of the study and the confidentiality of their responses. Thus, data collection was completely anonymous. Table 1 summarizes the two main sections in the questionnaire used and some of the questions that were asked.

Primary data were collected during May 2020, just a few months after the declaration of the national lockdown to contain the spread of the coronavirus in South Africa. As a result, data were collected by using an online platform and social media. Invitations to complete the questionnaire were sent to various WhatsApp, Telegram, and Facebook groups of which the first author was a member of these groups at the beginning of May 2020. This can be considered convenience sampling as discussed earlier. Members of these groups then shared the invitation with other groups and individuals and this method was reinforced by snowball or chain sampling. Snowball sampling is defined by Onwuegbuzie and Collins [40] as sampling where participants recruit other individuals of the same profile to join the study.

Data analysis was conducted by means of descriptive statistics to summarize the basic properties of the collected data. In addition, several inferential statistical tests were conducted. One of them was a bivariate analysis, which is an inferential statistical test to examine the relationships between two or more variables to determine the empirical association between them [42]. Three bivariate analyses were used to determine the relationship between recycling behavior and the socio-demographic variables of gender, age, education, and income. The independent sample *t*-test is used when a comparison is performed between the means of two groups to determine if there is a statistical difference between the groups [43], and in the present study, this was used for the variable of gender. For the variable of age, the Pearson correlation coefficient (*r*) was used to measure the strength and direction of the linear relationships between two quantitative variables and the degree to which the variables coincided with one another [44]. Analysis of variance (ANOVA) was used for the variables of education and income level. Such analysis is used when a comparison is made between group averages of a dependent variable across different levels of an independent variable [45].

Exploratory factor analysis (EFA) was used as a reduction method to help identify the drivers of recycling behavior. The statements that were used in this study are similar to those used in the study conducted by Tonglet et al. [46] to determine the drivers for households’ waste minimization behavior in Brixworth (UK). Thus, for the present study, 25 statements were used to determine the relevant factors which drive waste separation for recycling. The study did not investigate the intention to recycle; instead, the relationship between variables and the actual participation in recycling was explored. The statements used in the questionnaire were grouped under attitude, subjective norms, perceived control, moral norms, situational factors, consequences, and outcomes as previously indicated in Table 1. These statements were assessed by using a 5-point Likert scale that ranged from ‘strongly disagreeing’ to ‘strongly agreeing’ with them.

The calculated Cronbach alpha coefficient (α) was 0.97 and indicated a very good internal consistency [47,48]. The Kaiser-Meyer-Olkin (KMO) value was 0.956 (>0.6) and the value from the Bartlett’s Test of Sphericity (BTS) was 0.0 (*p* = 0.00 < 0.05). Thus, all the requirements were met to conduct exploratory factor analysis. The number of factors to retain and account for the correlations among the variables was determined by using the eigenvalue cut-off rule where only factors with values greater than 1.0 are retained, Catell’s scree test [47,48] and Horn’s parallel analysis [48,49]. Parallel analysis calculates the eigenvalues from a randomly generated dataset of the same size. Only factors whose eigenvalues exceeded the corresponding values from the random dataset were retained to represent drivers of recycling behavior. The next step was to rotate the factors for interpretation and the varimax rotation was used. Varimax rotation maximizes the sum of the variance of the squared loadings (correlations between variables and factors). According to Dilbeck [50], varimax rotation (also called Kaiser–Varimax rotation) is an attempt to clarify the relationship among factors. Furthermore, Brody [51] maintains that rotating a matrix allows for a full consideration of the trends, patterns, and themes that assist in the interpretation of the data. Furthermore, multiple regression analysis was used to predict values of a particular dependent variable which is recycling participation in this study. In this context, the independent variables were modeled as predictor variables [52]. Pederson [53] stated that multiple regression is often used when multiple factors contribute to explaining a particular phenomenon. Prior to conducting the multiple regression analysis, the assumptions of multiple regression were determined. The first requirement to be met was the sample size. The research work conducted by Tabachnick and Fidell [54] provided the formula for calculating the sample size requirements: N > 50 + 8 m (where m = the number of independent variables). Four independent variables were used in this study and therefore the required sample size was 82. With 398 respondents in this survey, this requirement was easily met. The second assumption that needed to be addressed in multiple regression is multicollinearity and which refers to a situation where the independent variables are highly correlated [48]. If multicollinearity is present, multiple regression cannot be performed as it undermines the statistical significance of an independent variable. The tolerance and VIF (variance inflation factor) values were used to check for multicollinearity. The cut-off points used were those suggested by Pallant [48]. Tolerance values of less than 0.10 and VIF values above 10 indicate multicollinearity. The tolerance value for each independent variable was >0.10 and the VIF values were below 10. Therefore, the multicollinearity assumption was not violated. 

### 2.3. Study Limitations

This study was constrained by a few limitations that are summarized as follows. Firstly, the study researched waste separation behavior and recycling drivers in the CoJ, the largest metropolitan complex in South Africa. However, South Africa can be considered “an unusual and extreme case in geography” [55] with unique territorial trends and urban settlements. Most urban settlements in South Africa range from metropolitan areas, secondary cities, and large towns to small towns that serve surrounding rural areas. There is therefore a high probability that the findings of this study would benefit other metropolitan areas and even secondary cities in South Africa. However, the findings may have limited generalizability to smaller towns as their settlement patterns and local government structures vary tremendously from those of large cities.

Secondly, the study approached potential respondents via social media communication platforms and this was followed by snowball sampling methods to broaden the sampling framework while online Google Forms conveyed the questionnaires. Whereas the surveys were able to collect a relatively large sample of data during a period of severe movement restrictions to contain the COVID-19 pandemic, using such a sampling strategy brings potential sources of bias. For example, individuals who were not digitally conversant or lacked social media affiliation at the time of the surveys could not participate, thereby unintentionally cutting out their households from the data collected. Similar study limitations have been reported in many online surveys undertaken during the COVID-19 lockdown periods [56,57,58]. In light of these limitations, caution is advised in the extrapolation of the present findings to all households in Johannesburg. 

Lastly, in terms of quantitative research surveys, a weakness identified by Onwuegbuzie and Leech [40] is the tendency that they represent only a snapshot of a dynamic phenomenon—in this case, we examined waste separation behavior during a specific period of time and in a restricted online context. 

## 3. Results

### 3.1. Demographic Characteristics of Respondents

In Table 2, the socio-demographical attributes of the respondents are indicated. To a large extent, most respondents were represented by women as they constituted nearly 80% (n = 314) of the whole sample whereas men amounted to only 20% (n = 80). Thus, women were highly over-represented in this study because, in the Gauteng province where the City of Johannesburg is located, women constitute only about 50% of the total provincial population [59]. This anomaly can be ascribed to the sampling strategy whereby WhatsApp and Telegram social groups with mostly female subscribers were consulted for the surveys, especially for the initial contacts with potential respondents. Two age groups constituted the highest proportions of respondents: 41–50 years (28%; n = 109) and 51–60 years (28%; n = 109), respectively. By contrast, other age groups of respondents were less represented. For instance, respondents aged 60 years and above amounted to nearly 10% (n = 38) out of the total number of respondents. In terms of educational achievements, a greater proportion (34%; n = 134) of respondents obtained post-graduate degree qualifications and this was closely followed by those with bachelor’s degree qualifications (26%; n = 104)) at the university level. However, about 20% of respondents completed high school studies with a national senior certificate. By income distribution, the highest proportion (28%; n = 110) of respondents were in the realized middle class (R300,001–R500,000); thus, nearly equal to approximately 27% of respondents whose income was in the emerging middle class (R100,000–R300,000). The lowest (9%; n = 34) proportion of respondents was found in the emerging income class.

### 3.2. Assessing Recycling Participation

The different statements on the degrees of participation in waste recycling amongst the respondents are shown in Table 3. Respondents who recycled everything that is recyclable amounted to 34% whereas those who recycled a lot but not everything constituted nearly 34%. On the other hand, the lowest (9%) proportion of respondents was comprised of those who do not recycle anything while those who recycled small quantities of materials constituted about 23% of the total. 

When all these percentages are considered together, the self-reported recycling participation rates were significantly higher (90%) than those reported in previous studies in the CoJ and official figures provided by both Pikitup and Statistics South Africa (StatsSA). For example, Pikitup [26] estimated the average participation rate in their S@S program to be around 20%, while the household survey by StatsSA provided a 31% recycling participation rate for Johannesburg [60].

### 3.3. Influence of Socio-Demographic Variables on Recycling Participation

An independent sample *t*-test was conducted to compare the recycling participation behavior against the gender of respondents. The test indicated no significant statistical differences between male (M = 2.81, SD = 0.995) and female (M = 2.94, SD = 0.979); *t* (392) = 1.033, *p* = 0.30) respondents. Thus, gender did not play a role in recycling unlike contrary results coming from previous studies [61,62,63,64]. 

The relationship between recycling participation and age was investigated by applying the Pearson product-moment correlation test. This test revealed a small but positive correlation between the two variables (*r* = 0.22, *n* = 397, *p* < 0.001) with older respondents associated with higher levels of participation in recycling. This outcome confirms that there is a statistically significant relationship between age and recycling participation in Johannesburg, thus in accord with the results of previous studies regarding the influence of age on recycling participation [65,66,67,68,69,70,71].

A one-way between-groups analysis of variance (ANOVA) was conducted to explore the effect of household income on levels of participation in waste separation. To achieve this goal, household incomes were subdivided into six different income levels (Table 2). The analysis revealed a statistically significant difference at the *p* < 0.05 level for the various income groups (*F* (5, 389) = 2.8, *p* = 0.014). However, the actual difference in mean scores between the income groups was quite small. Post hoc comparisons using Dunnett’s test indicated that the mean score for emerging affluent respondents (M = 2.53, SD = 0.929) was significantly different from affluent respondents (M = 3.22, SD = 0.759). 

Furthermore, education levels were divided into four different levels (Table 2). The results of the ANOVA showed no statistically significant difference in recycling participation and education level *F* (4, 397) = 0.787, *p* = 0.801. A possible reason for this can be that the majority (80.4%) of the respondents seem to be all educated, thus allowing for no significant difference between them except for those who were more highly educated than the rest of them.

### 3.4. Analyzing Variables related to Recycling Drivers

In this section, the results based on the descriptive statistics pertaining to the seven aspects of recycling drivers are summarized. For each aspect, namely attitude, subjective norms, perceived control, moral norms, situational factors, consequences, and outcomes, the results are discussed in the following sub-sections by using tables (Table 4, Table 5, Table 6, Table 7, Table 8 and Table 9).

#### 3.4.1. Attitudes

Four questions tested the attitudes of respondents towards separating waste for recycling. As shown in Table 4, on the whole, the majority of respondents displayed very positive attitudes towards household recycling. For instance, the statement that ‘recycling is useful’ exhibited the highest positive attitude as 90% of respondents were in agreement or strong agreement with it (Table 4). This was followed by ‘recycling is good’ as agreed/strongly agreed upon by 89% of respondents. By contrast, just over half (52%) of the respondents strongly agreed with the statement that ‘recycling is rewarding’. Such positive attitudes are critical for increased waste sorting at the household level. Similarly, the research reported by Tonglet et al. (2004) [46] indicated that positive attitudes amongst Brixworth (UK) residents were strongly associated with positive recycling intentions. 

#### 3.4.2. Subjective Norms

Compared with recycling attitudes, social pressure was not a very dominant driver of recycling participation (Table 5). Only about 26% of respondents expressed strong agreement with the statement ‘most people think I should recycle’ although moderate agreements were slightly higher (29%). By contrast, the social norm whereby people would approve of an individual who participates in recycling was highly agreed to by 36% of respondents, thus indicating the importance of the social pressure that individuals experience in society.

#### 3.4.3. Perceived Control

Perceived control over recycling participation was represented by 5 different statements. These statements included respondents’ perception of the ease of recycling, opportunities to recycle, and knowledge on how to recycle and what items can be recycled (Table 6). Being knowledgeable about what items are to be recycled (71%) was agreed/strongly agreed while 63% of respondents indicated they know how to recycle their household waste. Previous studies have demonstrated the positive influence of personal knowledge on pro-environmental behavior, including recycling behavior [9,72,73].

However, only 55% of respondents agreed with the statement that recycling is easy. This showed that even residents that were recyclers did not find it easy and more than a fifth (21%) of them indicated that there were not enough opportunities to participate in recycling.

#### 3.4.4. Moral Norms

The three statements that investigated the moral norms of respondents were ‘it would be wrong of me not to recycle’, ‘I feel I should not waste anything if it could be used again’, and ‘everybody should share the responsibility to recycle’ (Table 7). The levels of agreement were again lower than those of attitude towards recycling, but higher than those recorded for social norms. However, the proportion of respondents who expressed strong agreement with some of the statements was relatively higher. For instance, the percentage of respondents who strongly aligned with the statement ‘everybody should share the responsibility to recycle’ was relatively very high (53%), and this was followed in descending order by nearly 45% of respondents regarding the statement ‘it would be wrong of me not to recycle’.

#### 3.4.5. Situational Factors

The situational factors were the availability of time to separate wastes, storage of recyclables, and whether or not recycling is complicated (Table 8). The space required to store recyclables was an issue for 26% of respondents who indicated that recycling takes up too much space. For both ‘recycling takes up too much time’ and ‘recycling is too complicated’, nearly 30% of respondents were in strong disagreement. This outcome may be ascribed to high participation levels reported by respondents in this study, thus not unexpected because they are seemingly geared to undertake waste segregation at the home level.

#### 3.4.6. Outcomes

The outcomes tested were as follows: ‘recycling reduces pollution’, ‘saves landfill space’, ‘protects the environment’, and ‘preserves natural resources’ (Table 9). The highest level of agreement was recorded for ‘recycling helps to protect the environment’ (65% strongly agreed). For other outcomes, the proportions of respondents who were agreeing with them were slightly lower than the highest ones, thus pointing out the importance of these outcomes for recycling participation.

#### 3.4.7. Consequences

In Table 10, the results on several consequences that can be associated with recycling are exhibited. The following statements applied to these consequences: ‘recycling creates jobs’, ‘saves energy and money’, and ‘I can see the point in recycling’. A marked majority of respondents understood the importance of recycling as about 72% of them expressed strong agreement with the statement ‘I can see the point in recycling’. However, the level of agreement regarding the statement that ‘recycling saves money’ was supported by only 36% of respondents. 

### 3.5. Exploratory Factor Analysis Assessing Drivers of Recycling

As explained previously, the theory of planned behavior (TPB) maintains that individual behavior is driven by behavioral intentions. Such intentions are dependent upon three determinants, namely, attitude, perceived behavioral control, and subjective norms [46]. However, in the present research, behavioral intentions were not measured directly; instead, the role of other variables which may influence recycling behaviors was explored. Introducing such variables is consistent with conventional applications of the TPB model as it allows for the inclusion of additional factors [46,74], which were in the form of situational variables in the present study. 

In the present study, exploratory factor analysis and varimax factor rotation were performed to identify the underlying drivers of household waste separation behavior. Such analysis was based on the 25 statements summarized collectively in Table 1. The goal was to group the different variables coming from these statements into various constructs or factors. The dataset was suitable for factor analysis with more than 300 cases, and the Kaiser–Meyer–Olkin (KMO) statistic was 0.956, thus, exceeding the recommended value of 0.6 while Bartlett’s test of sphericity (BTS) test reached statistical significance (*p* = 0.00 < 0.05). Kaiser’s criterion, Catell’s scree test, and parallel analysis were used to determine the number of factors for consideration. The results of Kaiser’s criterion and parallel analysis revealed the presence of three main factors with eigenvalues exceeding 1 (Table 11). Therefore, a three-factor solution was adopted for analysis. 

The three factors represented separate and independent underlying dimensions of household waste separation behavior amongst the respondents and they explained about 68% of its variance. However, the outcome from the factor analysis did not group all the variables as previously shown in Table 1, which summarizes the questionnaire. This is because some of the theoretical factors were not particularly distinctive from one another, thus their effect was indistinguishable from one another. More particularly, the attitude and subjective norm items were too similar to justify them as different from one another. In the same way, the manner in which the residents responded to the various statements representing variables such as subjective norms, moral norms, benefits of recycling, attitudes, outcomes, and consequences was very similar. Hence, these variables were grouped together under a single factor that was named ′recycling benefits′.

In summary form, Table 12 indicates the different groupings under the three main factors as well as the individual factor loadings associated with each dimension. It can be seen that the first factor, named recycling benefits, has created a new structure to the existing TPB model. As explained previously, this factor derives from the combination of responses to all questions under (1) attitudes (including consequences and outcomes), subjective norms, and moral norms, (2) the positive aspects of perceived control, and (3) the negative aspects of perceived control: barriers. The second factor, perceived control, grouped four of the five perceived control statements—the exception was ‘recycling is inconvenient’ and it was subsequently grouped into the third factor. The third factor was comprised of situational variables and contained all the measures of situational factors as well as one measure of perceived control. Given the three main constructs or factors that were extracted from EFA, it is imperative to explain their importance further so that it can be understood how they influence household recycling behavior in the study area.

As depicted in Table 12, the recycling benefits construct was composed of 17 items that explained about 46% of the variance in recycling participation. The associated factor loadings ranged from 0.435 to 0.942. In light of this finding, it can be deduced that this factor strongly influences recycling behavior according to the different dimensions associated with it. Related studies conducted elsewhere have shown that waste recycling enhances environmental and societal benefits through the recovery of reusable materials and by limiting greenhouse gas emissions that would otherwise result from unrestrained waste landfilling [2,75,76]. The strongest item-to-factor loadings was recorded for ‘recycling helps to protect the environment’ and there was an excellent shared variance of approximately 89% (0.942) with this construct. Other important items were as follows, along with their shared variances: ‘recycling preserves natural resources’ (83%, 0.910), ‘recycling is good’ (79%, 0.890), ‘recycling is responsible’ (79%, 0.887), ‘recycling is useful’ (78%, 0.885), and ‘recycling saves landfill space (78%, 0.882). Given the dominant role of the recycling benefits factor in this analysis, the versatility of the TPB model to accommodate other determinants of behavior is demonstrated. However, more critically, communicating and sharing information about these benefits across the whole city (i.e., City of Johannesburg and households) is necessary to help transform some of their non-recycling practices into recycling ones; a point also raised by Vicente and Reis [77] in their research conducted in Portugal.

According to De Groot and Steg [78], perceived control has to do with a person’s belief regarding how easy or difficult it would be to perform a certain behavior, including recycling behavior in the context of the present study. The perceived control construct in the present study consisted of four items and collectively explained 12% of the variance with factor loadings ranging from 0.498 to 0.661. The item ‘recycling is easy’ (44%, 0.661) had a very good shared variance with the perceived control construct. Items such as ‘I have plenty of opportunities to recycle’ (39%, 0.624) and ‘I know how to recycle my waste (39%, 0.622) had a good shared variance as well, while the item ‘I know what items can be recycled’ (25%, 0.498) only had a relatively lower shared variance compared with other items. Based on these results, the individual capabilities and resources available to respondents have a positive influence on their recycling participation. Thus, the recycling behavior amongst residents can be maximized further if the city’s local municipality can create more opportunities, infrastructure, and other supporting resources to make recycling convenient for most households within their jurisdiction. 

Unlike the aforementioned factors, situational factors represent a person’s unique situation and derive from the various circumstances in which individuals find themselves temporally and spatially [79,80]. Furthermore, situational effects may influence other variables such as perceived control in the TPB model [81]. In the present study, the situational construct explained 11% of the variance in recycling participation and consisted of four items with factor loadings that ranged from 0.643 to 0.845. More specifically, three items had an excellent shared variance with the construct—‘recycling takes up too much time’ (71%, 0.845), ‘recycling takes up too much space’ (68%, 0.823), and ‘recycling is too complicated’ (53%, 0.727). The last item grouped under the situational variables factor was ‘recycling is inconvenient’, and it exhibited a very good shared variance of 41% (0.643). 

Given the results emanating from the exploratory factor analysis, it was necessary to determine further which of the three factors or constructs was exerting the greatest influence on household recycling behavior. To address this goal, a multiple regression analysis was performed using recycling participation as the dependent variable while the age of respondents, recycling benefits, perceived control, and situational variables were predictors of the desired behavior. The advantage of using multiple regression analysis is that it is capable of comparing the relative contribution of each predictor variable in explaining the variance in the desired behavior. This capability is performed by calculating the beta weight (β) which represents the various independent variables converted to the same scale [82]. The results are indicated in Table 13. It can be seen that the situational variables constituted the largest unique contribution (beta = 0.410, *p* < 0.001) to the prediction of recycling participation amongst the respondents, and this was followed by the role of perceived control (beta = −0.271, *p* < 0.001), age (beta = 0.141, *p* < 0.001), and recycling benefits (beta = −0.137, *p* < 0.05). In this way, the analysis has shown the comparative role of the different constructs explaining recycling participation in this study, over and above the results revealed by factor analysis alone. Lastly, the four variables considered in the multiple regression analysis collectively explained approximately 30% of the variance in recycling participation, thus revealing their overall impact on moderating behavioral outcomes in the study area.

## 4. Conclusions and Recommendations

As alluded to earlier in this paper, there are pressing waste management challenges in many developing countries, including South Africa, to move away from unsustainable traditional waste landfilling. Instead, a more sustainable approach is to adopt waste minimization strategies that can substantially reduce the increasing volumes of municipal solid wastes disposed of in landfill sites, thereby enhancing environmental protection, saving landfill space and natural resources, and re-purposing salvageable waste fractions back into the national economy. Therefore, the main goal of the present research was to assess the association between recycling behavior and socio-demographic variables for households in Johannesburg. The research also aimed at identifying the underlying driving factors that motivate recyclers to separate their household waste for recycling. This was performed within the expanded framework of the theory of planned behavior (TPB), with situational factors as an additional variable. This theoretical framework offers to identify key factors influencing household waste recycling behavior, thus yielding important lessons towards maximizing waste recycling behavior in the City of Johannesburg. The city has already embarked on household waste separation (for example, the S@S program) interventions although they are not unfolding successfully. To achieve the research objectives formulated in this paper, the study adopted a quantitative survey design, and data collected were analyzed by means of descriptive and inferential statistics. 

The majority of the respondents were female (79%), older than 40 years (65%), and 80% of them had completed at least a high school education qualification. The high preponderance of female respondents in this research is ascribable to the initial sampling framework which relied heavily on already established social media groups (for example, WhatsApp and Telegram) to recruit respondents into the questionnaire-administered online surveys. Despite potential biases, very important findings have been revealed, and unlike previous studies [2,4,56] conducted on household waste separation in South Africa, this study has exhibited relatively higher recycling participation rates amongst the respondents—with about 91% of them generally enlisted for separating waste at household level. However, only 34% of them regarded themselves as committed recyclers, thus suggesting the existence of recycling barriers to household recycling participation. 

A bivariate analysis was conducted to explore the relationship between four socio-demographic variables and recycling. The only variable that exhibited a statistically significant influence on recycling participation was age. Similarly, numerous studies have confirmed that age is a significant predictor of recycling participation rates in various urban settlements; for instance, in Minnesota counties (USA) and the Lombardy region (Italy) [66,83]. Moreover, a study by Martin et al. [65] found that in Burnley (UK), full recyclers were predominantly retired residents in households. It is therefore important to make household waste separation convenient to all age groups by removing the barriers confronting would-be recyclers so that they too can participate effectively without major impediments.

Regarding the aspects of attitude, subjective norms, perceived control, moral norms, situational factors, outcomes, and consequences of recycling which are some of the components of the theory of planned behavior (TPB) in this research, the respondents showed high levels of agreement with the various statements that investigated these aspects. However, the most positive aspect was the attitude of respondents towards recycling where almost three-quarters (74%) of them strongly agreed that ‘recycling is good’, followed by ‘recycling is useful’ (72%) and that respondents could see the point in recycling (72%). However, lower levels of agreement were found for situational factors, thus, suggesting that even recycling individuals were struggling with finding time and space to do this, apart from recycling being a complicated process. 

The exploratory factor analysis (EFA) was used to determine the number of factors that accounted for the pattern of correlations among the 25 statements that were presented to the respondents. To this extent, three factors were identified, namely recycling benefits, perceived control, and situational variables. However, the recycling benefits construct explained nearly 46% of the variance in the waste separation behavior, followed by the perceived control construct (12%), while the situational construct explained 11% of the variance. Lastly, the factors identified in the EFA as well as the age of respondents were subjected to a multiple regression analysis to determine which amongst the independent variables exerted the greatest influence on recycling participation. Although all four variables were found to be statistically significant and explained approximately 30% of the variance in recycling participation, the situational variables constituted the largest unique (beta = 0.410, *p* < 0.001) and relative contributions (7%) to recycling participation in the study area.

These findings have important implications for improved household waste separation programs in the City of Johannesburg and other metropolitan areas in South Africa with similar geographical features. On a pragmatic level, household waste separation schemes must be easier and user friendly to implement, especially from the perspective of residents who are expected to participate meaningfully in them. Therefore, it is very important to create awareness about recycling benefits in this city while making an effort to eliminate situational barriers such as the lack of waste-separation facilities in certain neighborhoods. In the same vein, both Vicente and Molina [72] maintain that better involvement in waste recycling schemes can be attained by demonstrating to people how their joint co-operative behavior can contribute to changing things (i.e., creating awareness about recycling benefits) and within their own perceived control. From the standpoint of future research work, similar research is highly recommended to reach as many types of residents as possible in the City of Johannesburg and related metropolitan areas in South Africa, thus drawing out important similarities and dissimilarities with the present research. 

## Figures and Tables

**Table 1:** A brief overview of the survey questionnaire.

Sections	Aspects
Section A	Demographical Aspects Questions were based on gender, age, education, and household income
Section B1	Testing recycling participation amongst respondents: Which statement best describes your recycling behavior? I recycle everything that can be recycled I recycle a lot but not everything I recycle small amounts I do not recycle
Section B2	Testing of the theory of planned behavior (TPB) by 25 statements arranged on a Likert scale * to measure recycling behavior (i.e., attitudes, subjective norms, perceived control, moral norms, situational factors, outcomes, and consequences). Statements on attitude: Recycling is good Recycling is useful Recycling is rewarding Recycling is responsible Statements on subjective norms: Most people think I should recycle Most people would approve of me recycling Statements on perceived control: Recycling is easy I have plenty of opportunities to recycle Recycling is inconvenient I know what items can be recycled I know how to recycle my waste Statements on moral norms: It would be wrong of me not to recycle I feel I should not waste anything if it could be used again Everybody should share the responsibility to recycle Statements on situational factors: Recycling takes up too much time Recycling takes up too much space Recycling is too complicated Statements on outcomes: Recycling reduces pollution Recycling saves landfill space Recycling helps to protect the environment Recycling preserves natural resources Statements on consequences: I cannot see the point in recycling Recycling saves energy Recycling saves money Recycling creates jobs

* The 5-point Likert scale ranged from ‘strongly disagreeing’ (1) to ‘strongly agreeing’ (5).

**Table 2:** Socio-demographical attributes of respondents.

Characteristics	Class	Frequency	Percentage
Gender	Female Male	314 80	78.9% 20.1%
Age group	≤20 years	5	1.3%
	21–30 years	49	12.3%
	31–40 years	87	21.9%
	41–50 years	109	27.5%
	51–60 years	109	27.5%
	60+ years	38	9.6%
Education	Up to Matric Post-matric diploma/certificate	78	19.6%
	Bachelor’s degree	81	20.4%
	Post-graduate degree	104	26.2%
		134	33.8%
Income	Lower (<R50,000 p/a) Emerging middle (R100,000–R300,000) Realized middle (R300 001–R500,000 Upper middle (R500,001–R750,000)	51	13.1%
	Emerging affluent	103	26.5%
	Affluent (>R750,000)	110	28.3%
		50	12.9%
		34	8.7%
		41	10.5%

**Table 3:** Statements on recycling participation rates amongst respondents.

Statements on Recycling Participation	Percentages (%)
I do not recycle	9.3%
I recycle everything that is recyclable	34%
I recycle a lot, but not everything	33.5%
I recycle small amounts	23.2%

**Table 4:** Attitude towards recycling.

Statements Expressing Attitude on a Likert Scale *	SA	A	N	D	SD
Recycling is good	74.3%	14.6%	2.3%	1.5%	7.3%
Recycling is useful	72.3%	17.9%	1.5%	1.0%	7.3%
Recycling is rewarding	51.1%	27.0%	13.1%	1.3%	6.5%
Recycling is responsible	69.5%	18.6%	3.0%	0.8%	8.1%

* The scale was subdivided as follows: (1) strongly agreed = SA, (2) agreed = A, (3) neutral, (4) disagreed = DA, and (5) strongly disagreed = SD).

**Table 5:** Subjective norms.

Statements Expressing Subjective Norms on a Likert Scale *	SA	A	N	D	SD
Most people think I should recycle	26.4%	29.0%	29.0%	8.8%	6.8%
Most would approve of me recycling	36.0%	33.2%	20.2%	4.8%	5.8%

* The scale was subdivided as follows: (1) strongly agreed = SA, (2) agreed = A, (3) neutral, (4) disagreed = DA, and (5) strongly disagreed = SD).

**Table 6:** Perceived control.

Statements Expressing Perceived Control on a Likert Scale *	SA	A	N	D	SD
Recycling is easy	23.9%	31.2%	18.9%	18.4%	7.6%
I have plenty of opportunities to recycle	28.6%	32.8%	17.8%	13.3%	7.5%
Recycling is inconvenient	29.7%	32.5%	19.6%	15.1%	3.1%
I know what items can be recycled	27.3%	44.1%	13.5%	7.0%	8.1%
I know how to recycle my waste	24.1%	40.2%	18.3%	9.8%	7.5%

* The scale was subdivided as follows: (1) strongly agreed = SA, (2) agreed = A, (3) neutral, (4) disagreed = DA, and (5) strongly disagreed = SD).

**Table 7:** Moral norms.

Statements Expressing Moral Norms on a Likert Scale *	SA	A	N	D	SD
It would be wrong of me not to recycle	44.8%	29.7%	11.8%	6.0%	7.6%
I feel I should not waste anything if it could be used again	41.6%	34.5%	13.1%	4.5%	6.3%
Everybody should share the responsibility to recycle	53.4%	31.2%	5.8%	2.3%	7.3%

* The scale was subdivided as follows: (1) strongly agreed = SA, (2) agreed = A, (3) neutral, (4) disagreed = DA, and (5) strongly disagreed = SD).

**Table 8:** Situational factors.

Statements Expressing Situational Factors on a Likert Scale *	SA	A	N	D	SD
Recycling takes up too much time	2.8%	13.1%	24.9%	29.5%	29.7%
Recycling takes up too much space	4.5%	21.7%	20.1%	29.5%	24.2%
Recycling is complicated	2.8%	15.9%	18.9%	32.7%	29.7%

* The scale was subdivided as follows: (1) strongly agreed = SA, (2) agreed = A, (3) neutral, (4) disagreed = DA, and (5) strongly disagreed = SD).

**Table 9:** Outcomes of recycling.

Statements Expressing Moral Norms on a Likert Scale *	SA	A	N	D	SD
Recycling reduces pollution	53.4%	28.7%	7.3%	3.3%	7.3%
Recycling saves landfill space	59.2%	23.7%	6.8%	2.8%	7.5%
Recycling helps to protect the environment	64.5%	22.4%	4.3%	1.3%	7.5%
Recycling preserves natural resources	62.0%	23.2%	6.0%	1.5%	7.3%

* The scale was subdivided as follows: (1) strongly agreed = SA, (2) agreed = A, (3) neutral, (4) disagreed = DA, and (5) strongly disagreed = SD).

**Table 10:** Consequences of recycling.

Statements Expressing Moral Norms on a Likert Scale *	SA	A	N	D	SD
I can see the point in recycling	71.5%	18.1%	7.3%	1.6%	1.5%
Recycling saves energy	44.6%	28.5%	15.4%	4.0%	7.5%
Recycling saves money	35.3%	30.5%	21.9%	6.0%	6.3%
Recycling creates jobs	50.9%	33.8%	6.5%	1.0%	7.8%

* The scale was subdivided as follows: (1) strongly agreed = SA, (2) agreed = A, (3) neutral, (4) disagreed = DA, and (5) strongly disagreed = SD).

**Table 11:** Calculated eigenvalues (EFA).

Factor	Initial Eigenvalues			Rotation Sums of Squared Loadings			Parallel Analysis
	Total	% of Variance	Cumulative %	Total	% of Variance	Cumulative %	Total
1	13.77	55.072	55.072	11.34	45.597	45.597	1.474455
2	3.25	12.992	68.064	2.94	11.747	57.344	1.410626
3	1.095	4.379	72.443	2.78	11.098	68.442	1.357274

**Table 12:** Factor analysis and factor loadings of each dimension.

Factors	Variables	Factor Loadings
Recycling benefits	Recycling is good	0.890
	Recycling is useful	0.885
	Recycling is rewarding	0.808
	Recycling is responsible	0.887
	People think I should recycle	0.519
	People would approve of me recycling	0.644
	It would be wrong of me not to recycle	0.659
	Not waste items that can be reused	0.676
	Everybody shares the responsibility	0.832
	Recycling reduces pollution	0.864
	Recycling saves landfill space	0.882
	Recycling protects the environment	0.942
	Recycling preserves natural resources	0.910
	I cannot see the point in recycling	0.435
	Recycling saves energy	0.770
	Recycling saves money	0.668
	Recycling creates jobs	0.866
Perceived control	Recycling is easy	0.661
	I have plenty opportunities to recycle	0.624
	I know what items can be recycled	0.498
	I know how to recycle my waste	0.622
Situational variables	Recycling is inconvenient	0.643
	Recycling takes up too much time	0.845
	Recycling takes up too much space	0.823
	Recycling is too complicated	0.727

**Table 13:** Calculated coefficients and correlations statistics.

	Unstandardized Coefficient B	Std. Error	Standardized Coeff. Beta	*t*	Sig.	Correlations Zero-Order	Partial	Part
(Constant)	2.326	0.275		8.457	0.000			
Age	0.011	0.003	0.141	3.270	0.001	0.212	0.163	0.139
Recycling benefits	−0.137	0.062	−0.137	−2.214	0.027	0.152	−0.111	−0.094
Situational variables	0.372	0.060	0.410	6.218	0.000	0.422	0.300	0.264
Perceived control	−0.280	0.049	−0.271	−5.726	0.000	−0.420	−0.278	−0.243

## Data Availability

Data are available upon request.
